# Optimization of Tissue Digestion Methods for Characterization of Photoaged Skin by Single Cell RNA Sequencing Reveals Preferential Enrichment of T Cell Subsets

**DOI:** 10.3390/cells13030266

**Published:** 2024-01-31

**Authors:** Terri Clister, Rosalyn M. Fey, Zachary R. Garrison, Cristian D. Valenzuela, Anna Bar, Justin J. Leitenberger, Rajan P. Kulkarni

**Affiliations:** 1 Department of Dermatology, Oregon Health & Science University, Portland, OR 97239, USA; clister@ohsu.edu (T.C.); feyr@ohsu.edu (R.M.F.); garrisoz@ohsu.edu (Z.R.G.); bara@ohsu.edu (A.B.); leitenbe@ohsu.edu (J.J.L.); 2 Department of Surgery, Oregon Health & Science University, Portland, OR 97239, USA; valenzcr@ohsu.edu; 3 Cancer Early Detection Advanced Research Center (CEDAR), Portland, OR 97239, USA; 4 Knight Cancer Institute, Oregon Health & Science University, Portland, OR 97239, USA; 5 Operative Care Division, U.S. Department of Veterans Affairs Portland Health Care System, Portland, OR 97239, USA

**Keywords:** tissue digestion, scRNA-seq, skin cancer, melanoma, immune cell composition, T cells

## Abstract

Healthy human skin tissue is often used as a control for comparison to diseased skin in patients with skin pathologies, including skin cancers or other inflammatory conditions such as atopic dermatitis or psoriasis. Although non-affected skin from these patients is a more appropriate choice for comparison, there is a paucity of studies examining such tissue. This lack is exacerbated by the difficulty of processing skin tissue for experimental analysis. In addition, choosing a processing protocol for skin tissue which preserves cell viability and identity while sufficiently dissociating cells for single-cell analysis is not a trivial task. Here, we compare three digestion methods for human skin tissue, evaluating the cell yield and viability for each protocol. We find that the use of a sequential dissociation method with multiple enzymatic digestion steps produces the highest cell viability. Using single-cell sequencing, we show this method results in a relative increase in the proportion of non-antigen-presenting mast cells and CD8 T cells as well as a relative decrease in the proportion of antigen-presenting mast cells and KYNU^+^ CD4 T cells. Overall, our findings support the use of this sequential digestion method on freshly processed human skin samples for optimal cell yield and viability.

## 1. Introduction

The skin is the human body’s major interface with the external environment and as such is subject to numerous challenges, including pathogen exposure, sun exposure, physical injury, and environmental toxins. In addition to acting as a physical barrier which defends against pathogens, the skin contains a diverse resident immune cell population that serves a critical role in immune response and homeostasis [1,2,3]. Among the immune cells resident in the skin, T cells are important for pathogen defense, managing inflammation, and maintaining homeostasis, but upon dysregulation, they can also contribute to inflammatory skin conditions like psoriasis and atopic dermatitis [4,5]. Skin cancers, including both melanoma and non-melanoma skin cancers, represent another case in which there may be dysregulation of the resident immune cell population [6,7].

T cells can also play a role in the pathogenic dysregulation of skin homeostasis during cancer treatment. Many advanced cancer patients receive immune checkpoint blockade (ICB) therapy, and up to 90% of these patients are likely to develop immune related adverse events (irAEs)—auto-immune-like manifestations which result from the body’s response to immune cell activation [8,9,10]. Although irAEs manifesting in the skin have been widely studied, skin from healthy donors is normally used as a control for comparison to irAE skin from cancer patients. However, patients with skin cancer may have skin more appropriately described as sun-exposed or even pre-malignant. To effectively understand the changes that happen in the skin of patients undergoing ICB therapy for skin cancer, we must describe the baseline state of the resident immune cell population in the skin of similar patients prior to ICB therapy and irAE development.

The composition of healthy skin’s resident immune cell population has been described using flow cytometry [11] and fluorescent-activated cell sorting (FACS) followed by bulk transcriptome analysis [12] as well as using single-cell RNA sequencing (scRNA-seq), where the choice of tissue digestion method can greatly affect the reported results [13,14,15,16]. Studies in the gut and muscle tissue have also reported that the cell population profile varies depending on the digestion method used [17,18]. This issue may be exacerbated by the variable amount of connective tissue present in skin, rendering dissociation a challenging task which often produces few cells [3,13]. Several studies have produced protocols with variable parameters to address this issue. He et al. compared cryopreserved human skin tissue, used for most of the study, to a single freshly processed skin sample [19], while Sato et al. paired an enzymatic digestion with a scraping- or a migration-based isolation to compare the number and characteristics of isolated T cells [20]. Thus, although some progress has been made, the field lacks a comparison between methods to assess the importance of several key parameters to maximize cell yield and viability. 

To help address this need, we optimized and compared several methods for isolating single immune cells from human skin specimens. Importantly, we particularly focused on analyzing sun-exposed and sun-damaged skin of patients who were undergoing surgeries for the excision of melanoma or non-melanoma skin cancers; these tissues may be considered “pre-malignant” and at risk for development of cutaneous malignancy. 

We examined both freshly collected and viably frozen skin tissues. Patient samples cannot always be processed directly after collection, and many tissue archives may have viably frozen samples available for analysis. Therefore, the investigation of methods allowing for appropriate processing and analysis of such samples, and understanding limitation in methodology and changes to relative populations of cells recovered will be of benefit to the field.

We compared the viability and yield/number of cells extracted from fresh and frozen surgically resected skin sections using three different digestion methods. We found that tissue dissociation using two sequential enzymatic digestion steps resulted in higher cell viability and yielded greater numbers of cells per gram of tissue. Additionally, we used scRNA-seq to explore the impact of tissue digestion method on immune population and observed significant changes in the relative proportions of mast cells and T cells recovered from skin tissue. 

## 2. Materials and Methods

### 2.1. Collection of Non-Affected Skin from Skin Cancer Patients

We collected non-affected surgically resected skin tissue from 5 patients diagnosed with melanoma or non-melanoma skin cancers. One of the patient samples was a large section with enough cells after digestion to divide into three cryovials prior to freezing. We considered these as 3 separate samples when comparing cell counts and viability after thawing. These samples were collected over the course of two years, digested on the day of collection, and viably frozen at −150 °C. The frozen cells were not immediately analyzed due to institutional personnel and research restrictions during the COVID pandemic. When the first samples were thawed and observed to have suboptimal recovery of frozen cell number and viability, we decided to compare frozen and freshly digested samples. Like the frozen samples, fresh skin samples of standing cones and extra marginal skin adjacent to surgical excision were collected from dermatology surgical patients. Samples for fresh analysis were collected from a total of 8 patients. The fresh skin samples were large enough to make multiple 4 mm sized sections and test different digestion methods on a single patient’s skin sample. Of the 8 skin tissues collected (1 intact tissue sample from each patient), 3 had sufficient amount to be divided into 3 sections and digested using all three digestion methods discussed below; 4 tissue samples were divided into 2 sections and digested with the simultaneous and sequential method; and 1 patient’s tissue sample was digested with only the overnight method. 

We consider the skin samples used in this work non-affected rather than healthy, as the donors have been diagnosed with skin cancer and due to field effects, the neighboring skin likely has pre-malignant changes. Indeed, several patient specimens were specifically noted to have damaged or aged skin. The state of the non-affected tissue makes this dataset a prime resource for exploring the baseline state of the skin of skin cancer patients prior to surgical resection and/or ICB therapy. 

### 2.2. Tissue Digestion and Cell Counting

Three different tissue digestion methods were used: simultaneous digestion, sequential digestion, and overnight digestion. For each digestion method, as much visible fat is removed as possible from the tissue section, it is weighed in a petri dish and then cut into smaller parts (finely minced for the simultaneous and overnight digestion and cut into 2 mm pieces for the sequential digestion) while submerged in the respective digestion buffers.

The sequential digestion’s initial buffer contains dispase II (10 mg/mL, Sigma, St. Louis, MO, USA) in RPMI with 10% FBS. The tissue is incubated with shaking (800 rpm) at 37 °C for 45 min. Undigested tissue is further minced in a petri dish; then, it is returned to a 50 mL falcon and pelleted. The dispase buffer is removed and replaced with RPMI/10% FBS containing liberase (0.5 mg/mL, Liberase TL, a propriety blend of collagenase I and collagenase II, Roche, Mannheim, Germany) and DNase (50 U/mL, Roche). The sample is again incubated with shaking at 800 rpm, 37 °C for 45 min. The digested cells are filtered through a 40 µm strainer, and the strainer washed at least 3 times with additional media. The cells are pelleted and if the pellet is contaminated by red blood cells, they are lysed using 1× RBC lysis buffer (eBioScience, Invitrogen, Carlsbad, CA, USA) according to the manufacturer’s protocol. Cells are resuspended in buffer in preparation for cell counting and when applicable, CD45^+^ isolation.

The simultaneous digestion buffer contains DNase I (50 U/mL, Roche), Hyaluronidase (600 U/mL, Worthington, Lakewood, NJ, USA), and collagenase IV (600 U/mL, Worthington) in DMEM/F12 media. Once minced in digestion buffer, the tissue and media are transferred to a 50 mL falcon tube and allowed to digest for 2 h at 37 °C, shaking at 800 rpm. After incubation, the undigested tissue is filtered out using a 100 µm strainer, and the digestion tube is washed with an additional 5 mL of DMEM/F12 and then combined with the digested tissue. Cells are pelleted at 1870 rpm for 10 min. If the pellet is contaminated by red blood cells, they are lysed using 1× RBC lysis buffer using the manufacturer’s protocol. The remaining cells are resuspended in freezing media (FBS/10% DMSO) and viably frozen in a Mr. Frosty at −80 °C before transferred to liquid nitrogen (−150 °C) for long-term storage or resuspended in buffer in preparation for cell counting and, when applicable, CD45^+^ isolation.

The overnight digestion method is based on a published skin digestion method used for single-cell sequencing [21]. The overnight digestion buffer is RPMI containing collagenase IV and DNase I. Samples may be stored in PBS at 4 °C up to 8 h before beginning digestion. Once minced in digestion buffer, in the same buffer, tissue is incubated at 37 °C, without shaking, overnight (16–18 h). Samples are then filtered through a 100 µm strainer, and the strainer is washed with additional RPMI. Cells are pelleted, buffer is removed, and the sample is resuspended in the buffer for cell counting and CD45^+^ isolation.

For all digestion methods, after the final resuspension, Calcein AM (2 mM, Invitrogen, Eugene, OR, USA) and DRAQ7 (BD, San Diego, CA, USA) were added at a 1:200 ratio to stain live and dead cells. The cell mixture was heated at 37 °C for 5 min then placed on ice. Cells were counted using the hemocytometer function on the BD Scanner to determine the number of cells and cell viability.

### 2.3. Sample Pooling and Labeling

Frozen cryovials of dissociated skin cells from standing cones were thawed and cells are transferred to warm DMEM/F12 media pre-aliquoted in 15 mL falcon conical vials. Cells were pelleted at 1870 rpm for 10 min. Fresh or frozen cells from patients were sequentially labeled with sample tag (BD, an oligo that labels a cell’s plasma membrane, enabling distinction between samples in the pooled single-cell data), FC block (BD), and 33 AbSeq antibodies (BD; antibodies against specific surface antigens that also have an oligo tag that will be isolated and amplified during cDNA preparation, as listed in Table S1). After labeling, cells were washed once and resuspended in buffer to begin CD45^+^ isolation.

### 2.4. CD45^+^ Cell Isolation

CD45^+^ cells were isolated from total skin cells using Miltenyi Biotech CD45^+^ TIL REAlease isolation kit, according to the manufacturer’s protocol. Briefly, cells were stained with a CD45-binding biotin-containing proprietary release compound that was then bound by an anti-biotin microbead. All cells were flowed through an MACS MS column with the CD45^+^ cells magnetically bound in the column. The column was rinsed and then removed from the magnet, cells labeled with the CD45^+^ antibody were pushed out of the column, and the CD45^+^ marker was removed. The initial flow through and washes were collected as the CD45^−^ fraction. Following CD45^+^ isolation, scRNA-seq was performed using the BD Rhapsody platform, following the manufacturer’s protocol, as outlined below.

### 2.5. Single-Cell RNA-Seq Cell Isolation and Library Preparation

Cells were labeled (or re-labeled) with DRAQ7 and CalceinAM to label live and dead cells. A BD Scanner was used to count cells and cell viability as well as calculate sample pooling. Cells were pooled such that all cells or a total estimate of 60,000 cells would be loaded onto a BD Cartridge. Cells and beads were loaded onto the cartridge according to the manufacturer’s protocol, with a 20-min wait time to let cells settle into individual wells prior to the addition of beads. cDNA was made and amplified according to the manufacturer’s protocol using the protocol for WTA (whole transcriptome analysis), SMK (sample multiplexing kit), and AbSeq library preparation. Completed paired-end 150 base-pair libraries were sequenced on a NovaSeq 6000 sequencer (Novogene, Sacramento, CA, USA).

### 2.6. Single-Cell RNA-Seq Data Analysis

Data preprocessing was performed using the Seven Bridges BD Rhapsody Whole Transcriptome Analysis Pipeline. Briefly, samples were demultiplexed and reads were quality filtered and aligned to the hg38/GRCh38 genome prior to quantification. Analysis was implemented using Seurat version 4.4.0 [22]. Quality filtering was performed to exclude cells expressing less than 200 unique transcripts and with greater than 25% mitochondrial transcript expression, and the CD45^+^ and CD45^−^ fractions were analyzed separately. The CD45^+^ datasets were integrated using the VST method, and the clustree [23] software version 0.5.0 was used to guide the choice of clustering resolution prior to visualization with uniform manifold approximation and projection (UMAP) [24]. Clusters were annotated using classic marker genes as well as selected genes from the literature [25,26,27] (Figure S1). Red–green colorblind-friendly visualization was performed with dittoSeq [28], and the R package scProportionTest [29] was used to compare relative proportions of cell types between digestion methods. Custom code used to analyze these data is available at https://github.com/rfey/skin-digestion (created on 30 November 2023).

## 3. Results

### 3.1. Fresh Tissues Have Higher Yield of Cells, Though Frozen Dissociated Tissues Have Acceptable Viability and Yield

**Figure 1:**
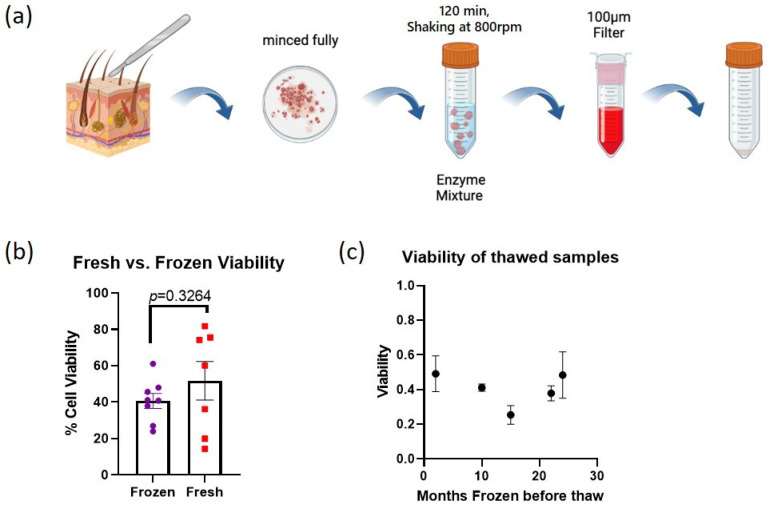
Comparison of fresh and frozen cell viability using the simultaneous digestion method. (**a**) Graphical depiction of the simultaneous skin digestion method. (**b**) Comparison of the average percent cell viability (# live cells/# total cells) between fresh (N = 7) and frozen samples (N = 8). (**c**) Relationship between months stored at −150 °C and the cell viability upon thaw (averages of two technical replicates are shown). Error bars indicate SEM.

The original digestion method we utilized, which we have termed the “simultaneous” digestion method, originates from a tumor digestion protocol optimized for increased cell yield [30]. In this method (Figure 1a), the tissue is dissociated in a single incubation step with one enzyme cocktail of collagenase IV, Hyaluronidase, and DNAse I in DMEM/F12 media, and the minced tissue section is digested for two hours before filtering for the final cell product. All frozen samples were dissociated into single cells using this method. They were then immediately viably frozen and stored at −150 °C for between three months and two years before further processing. As these samples were thawed for further analysis, we noticed suboptimal cell recovery in number and lower than expected viability. We decided to directly compare the “simultaneous” digestion method between previously frozen samples and fresh samples. Ultimately, two additional digestion methods were tested on fresh tissue and will be discussed in a later section.

There is a clear difference in percent cell viability (# live cells/# total cells) between the eight thawed samples (40.7% ± 4.2) and seven freshly digested samples (51.7% ± 10.6) (Figure 1b). Due to variability between patient samples, although there is a strong trend demonstrating the substantially better viability of non-frozen cells, it is not statistically significant (unpaired *t*-test, *p* = 0.3264). Interestingly, there was little relationship between the length of cryo-storage and cell viability upon thaw (Figure 1c). Unsurprisingly, these results indicate that even a short (3 month) cryostorage of viably frozen skin cells leads to noticeable loss compared to fresh cells isolated from skin using the same digestion method.

We also performed single cell RNA-seq on fresh and frozen tissue samples. As the quality control metrics were substantially worse for frozen tissue compared to fresh (Figure S2), we proceeded to test digestion protocols on fresh tissue only.

### 3.2. Fresh Tissue Digestion Optimization

#### 3.2.1. Methods for Optimization

**Figure 2:**
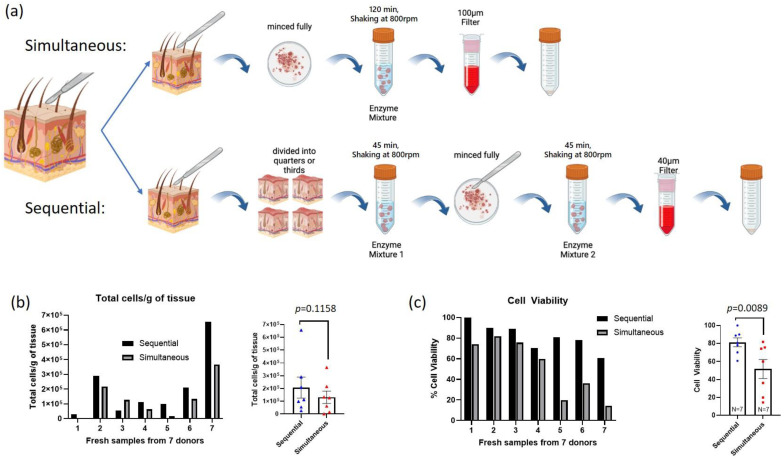
Comparison of cell isolation counts and viability between sequential and simultaneous digestion methods on fresh skin samples. (**a**) Graphical comparison of the simultaneous and sequential digestion methods. (**b**) Total cells/g of tissue isolated from the same patient using either the sequential or simultaneous digestion method (left). The average across 7 samples is also shown (right, N = 7, paired *t*-test, difference not significant). (**c**) Separated by patient (left) and average (right, N = 7, paired *t*-test *p* = 0.0089) percent cell viability (# live cells/# total cells) between sequential and simultaneous digestion methods. Error bars show SEM.

To further test the importance of digestion method on fresh skin analysis, we compared the previously described simultaneous method with two other methods adapted from the recent literature (Figure 2a and Figure S3). Each of these methods uses a unique enzyme combination and digestion time for human skin samples, which yields high cell counts and viability for scRNA-seq; these three were chosen based on the original reported yields, the commercial availability of the enzymes needed, and the relative minimization of ‘hands-on’ processing time.

The first, “simultaneous” method was described previously and used to compare fresh and frozen skin samples. In the second approach, called the “sequential” digestion method, the tissue is cut into quarters or thirds (depending on the size of the biopsy) rather than fully minced; then, it is incubated with dispase dissolved in RPMI media with 10% FBS for 45 min, which was followed by a second 45-min incubation with liberase (a proprietary blend of collagenases, see Methods for details) and DNAse I in RPMI/10% FBS media. After the second 45-min incubation, the tissue is filtered and spun for the final cell product (Figure 2a). This method was previously optimized for single-cell sequencing and has a smaller filter size to better isolate single cells [31]. 

In the “overnight” digestion method, adapted from a recent study examining immune populations in inflamed skin [21], the tissue is allowed to digest overnight in an incubator at 37 °C, 5% CO_2_; submerged in an enzyme cocktail of collagenase IV and DNAse I in RPMI/10% FBS media. Importantly, the tissue is stationary during overnight incubation rather than being on a shaking incubator. The following morning, the tissue is shaken by hand for 30 s before filtering and spinning for the final cell product.

#### 3.2.2. Sequential Digestion Has Greatest Cell Yield and Viability

We began by measuring the post-digestion cell viability (number of live cells/total number cells) for fresh skin samples with the goal of determining which digestion method best preserves cell viability and cell populations of skin. Standing cones from six patients undergoing dermatological surgery were divided into two or three 4 mm sections with one section each being used for simultaneous (N = 7), sequential (N = 7), and if enough tissue was available, overnight (N = 3) digestion methods. An additional standing cone was digested with the overnight method only. In this way, we are able to compare the digestion approaches within the same patient. 

Initial viability measurements showed that the sequential digestion protocol consistently yielded the greatest number of high viability cells (Table S2, Figure 2b,c). We compared both the percent cell viability and total number of cells isolated (live and dead) per gram of tissue between the different methods. Total cells per gram of tissue can be affected by the skin sample composition (i.e., fat content), so we do not have an empirical number of cells we expect per gram of tissue, but it provides a way to compare the number of cells isolated using different methods from the same tissue, and to a lesser degree, between patient samples. Across the six tissue samples large enough to section and digest with multiple methods, there is a fair amount of variability in the total cells isolated per section, ranging, for example, from 0.2 × 10^5^ to 3.2 × 10^5^ live cells/g of tissue for the sequential digestion method (Table S2). Six of the seven tissues had more live cells extracted using the sequential method compared to the simultaneous method (Figure 2b). However, one tissue had substantially more cells isolated using the simultaneous method instead. Therefore, while there is not a statistically significant difference in the number of total cells per gram of tissue isolated between the sequential and simultaneous digestion methods (paired *t*-test, *p* = 0.1158), there is a clear trend of more cells isolated using the sequential method (six of seven samples tested) and consistently better viability for all seven skin samples directly compared. 

Across all seven skin samples split and digested with both the sequential and simultaneous digestion methods, the single-cell viability is better using the sequential method (Figure 2c). On average, skin digested using the sequential method (separate digestion steps: dispase followed by liberase and DNAse I) yielded the highest consistent average cell viability at 81.2% ± 4.97 SEM compared to the simultaneous two-hour digestion with collagenase IV, hyaluronidase, and DNAse I (51.7% ± 10.57 SEM) and the overnight digestion with collagenase IV and DNAse I (38.98% ± 16.4 SEM) (Figure 2c and Figure S3b). There is a statistically significant difference in cell viability between the sequential and simultaneous digestion methods (paired *t*-test, *p* = 0.0089). There is also a clear decrease in cell viability using the overnight method compared to the sequential method. Both a paired *t*-test using three patient samples split between digestion methods and unpaired *t*-test using all digested samples (including those patients digested only with either the sequential or overnight method) showed a significant difference between the sequential and overnight digestion cell viability (paired *t*-test, *p* = 0.0364 or unpaired *t*-test, *p* = 0.0129) (Figure S3c). The overnight digestion had notably worse yield and overall viability on the three samples with paired simultaneous and sequential digestions (15.2% overnight vs. 100% sequential, 38.4% overnight vs. 80.6% sequential, and 16.8% overnight vs. 78.0% sequential). The additional patient that had tissue digested with the overnight method had much better viability, which was closer to the sequential methods (85.6% viability).

Given the consistently better cell viability and total cell counts we obtained for the sequential digestion method, we concluded this is the best overall method for single-cell dissociation of skin tissue. Next, the specific immune cell composition across the different digestion methods was compared using single-cell sequencing to better understand how the digestion methods preferentially isolate different immune cells present in non-affected skin.

### 3.3. CD45^+^ Immune Cell Enrichment

**Figure 3:**
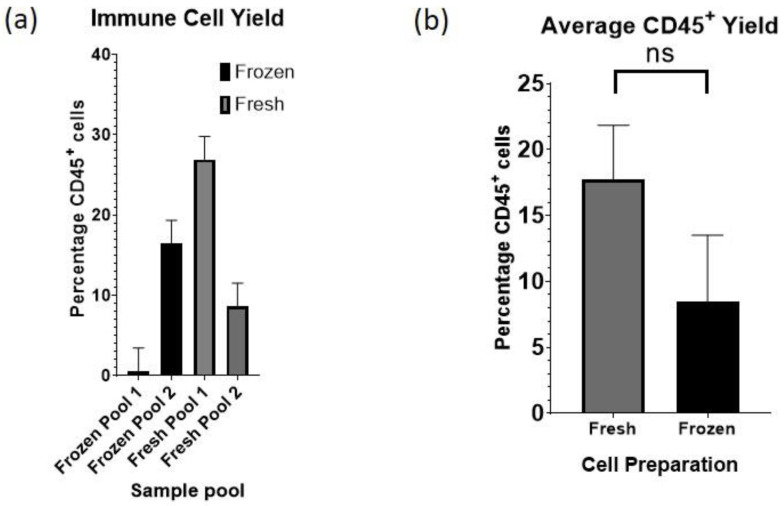
Immune cell yield of fresh vs. frozen skin samples. (**a**) Percentage of CD45^+^ cells enriched from skin tissue, four sets of pooled samples enriched separately. (**b**) Average proportion of CD45^+^ cells enriched from fresh or frozen cell samples. Error bars show SEM.

Skin immune cell activity and immune surveillance is increasingly recognized as critical both in the normal physiology and in pathological conditions that can be seen within skin. Given the paucity of research establishing the proportion of immune cells within the non-cancerous skin of skin cancer patients, we set out to enrich and identify their proportion relative to the total cell population. To collect as many immune cells as possible, we decided to focus on all CD45^+^ cells. We used positive enrichment (see Section 2 Methods for details) to isolate the CD45^+^ cell population from total skin cells recovered from both frozen samples (isolated with the simultaneous digestion) and fresh samples (digested with either the simultaneous, sequential, or overnight method). In preparation for scRNA-seq, five frozen and four fresh individual patient samples were tagged for multiplexing and combined into four pooled samples (two frozen and two fresh pools) prior to CD45^+^ isolation via bead column (Miltenyi). We found that the average proportion of CD45^+^ cells in each of the four pooled samples varied widely (0.5–27%, Figure 3a), which may be a function of the specific body area from which they were isolated, the underlying variability among patients, and/or any subclinical inflammation due to damage inherent to the sun-exposed locations from which they derived. While the average percentage of CD45^+^ cells recovered was double in freshly processed cells (17.75% ± 2.89 SEM) compared to frozen cells (8.49% ± 3.55 SEM) (Figure 3b), the difference was not statistically significant (unpaired *t*-test, *p* = 0.1802).

### 3.4. Single-Cell RNA-Seq Reveals Differential T Cell Clusters in Photoaged Skin 

Finally, we sought to determine whether these digestion methods are appropriate for preparing fresh skin tissue for single-cell sequencing. We performed paired single-cell RNA-seq and protein profiling to compare the effect of digestion methods on the composition of the immune cell population in the non-affected skin of skin cancer patients prior to ICB therapy. Briefly, cells from each sample were tagged with a sample multiplexing kit that uses an oligo-tagged antibody targeted to bind all cells (BD). The oligo from the sample tags is amplified during library preparation and with the associated cell label, it is used to distinguish cells from each sample. The uniquely labeled cell samples were pooled and sorted into CD45^+^ and CD45^−^ fractions prior to single-cell sequencing using the BD Rhapsody well-based system. Due to the lower quality sequencing results obtained with the frozen samples (Figure S2), we performed further analysis with results from the fresh samples only. Data analysis was performed separately for each fraction (CD45^+^ and CD45^−^), as described in the Methods Section. 

The initial evaluation of quality control metrics revealed little difference in the percentage of mitochondrial genes, the number of molecules, or the number unique genes detected in the CD45^−^ fraction. In the CD45^+^ fraction, we observed a slightly lower number of molecules and number of unique genes detected, and there was a higher percentage of mitochondrial genes in the sequential digestion method compared to the simultaneous method (Figure S4). Of note, while tissue digested using the overnight method was submitted for sequencing, no cells were recovered, and thus we have analyzed the simultaneous and sequential tissue digestion methods only using scRNA-seq.

**Figure 4:**
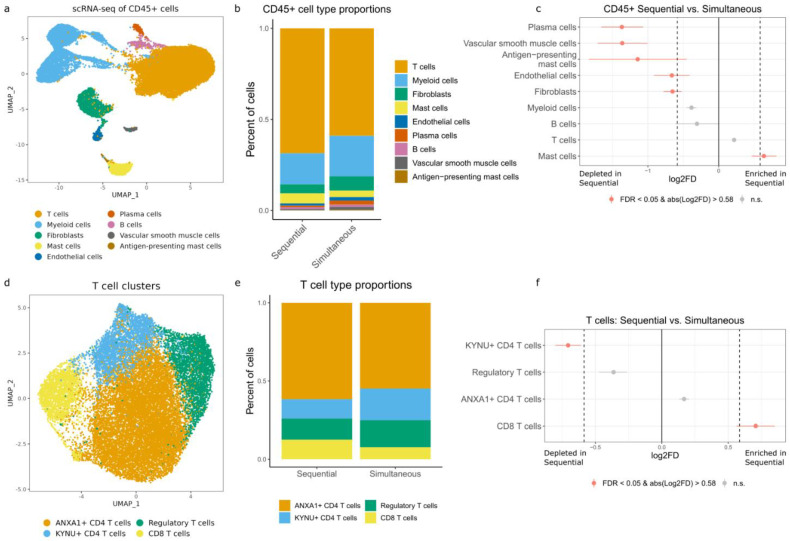
Single-cell RNA-seq analysis of CD45^+^ cells isolated from fresh tissue samples. (**a**) UMAP plot of 29,647 annotated CD45^+^ cells colored by cell type. (**b**) Stacked bar plot showing the relative proportions of each CD45^+^ cell type split by digestion method. (**c**) Relative differences in cell proportions for each CD45^+^ cluster, comparing sequential and simultaneous digestion methods. Red clusters are statistically significant (FDR < 0.05 and absolute log_2_ fold change > 0.58). Larger log_2_ fold changes indicate a higher proportion of cells in the sequential method. (**d**) UMAP plot of 19,357 T cells colored by cell type. (**e**) Stacked bar plot showing the relative proportions of each T cell type split by digestion method. (**f**) Relative differences in cell proportions for each T cluster, comparing sequential and simultaneous digestion methods. Red clusters are statistically significant (FDR < 0.05 and absolute log_2_ fold change > 0.58). Larger log_2_ fold changes indicate a higher proportion of cells in the sequential method.

After quality filtering and data analysis, we identified nine major cell types in the CD45^+^ fraction (Figure 4a), the majority of which were myeloid (N = 5574) and T cells (N = 19,357). To determine if any cell types were preferentially enriched or depleted for each digestion method, we visualized the proportion of each cell cluster on a stacked bar plot split by digestion method (Figure 4b) and performed a statistical proportion analysis for each cluster. We examined clusters with significant (FDR < 0.05 and log_2_ fold change > 0.58, corresponding to a fold change of 1.5) differences between the sequential and simultaneous digestion methods (Figure 4c, Table S3), and we found changes in two mast cell populations. Antigen-presenting mast cells were relatively depleted with the sequential method (0.38% in simultaneous, 0.17% in sequential, FDR = 0.001), while non-antigen-presenting mast cells showed an increased proportion (3.53% in simultaneous, 5.49% in sequential, FDR = 0.001). Additionally, plasma cells showed a significant decrease (2.15% in simultaneous, 0.83% in sequential, FDR = 0.001).

As T cells are known to play a critical role in skin resident immunity and were the largest group of cells in the CD45^+^ fraction, we next investigated T cell population changes dependent on digestion method. We identified four T cell subclusters (Figure 4d) and performed statistical proportion analysis as above. We found a significant increase in CD8 T cells in the sequential method (7.70% in simultaneous, 12.57% in sequential, FDR < 0.001) and a significant decrease in KYNU^+^ CD4 T cells (20.18% in simultaneous, 12.37% in sequential, FDR < 0.001) (Figure 4e,f, Table S4).

For the CD45^−^ fraction, we identified eight major cell types (Figure S5a), the majority of which showed no change depending on digestion method (Figure S5b). A statistical proportion analysis revealed a significant increase in melanocytes (0.59% in simultaneous, 1.04% in sequential, FDR = 0.003, Figure S5c, Table S5).

## 4. Discussion

We have compared three different tissue digestion methods for single-cell sequencing and analysis of immune and non-immune cells from human skin; these three methods were selected based on prior reports from the literature. Previous reports have shown mixed results on comparisons of fresh vs. frozen tissue cell viability, counts, and scRNA-seq expression, likely due to differences in tissue type, and dissociation or the cryopreservation method [15,32,33]. We showed here that fresh skin samples tend to result in higher cell viability compared to skin cells which have been frozen and subsequently thawed. We find that the sequential enzyme processing of fresh human skin samples tends to result in a higher number of cells per gram of processed tissue, and it produces a higher percentage of cell viability compared to the simultaneous digestion method. Taken together, these results suggest that the sequential enzymatic processing method used on fresh tissue is the optimal pipeline for processing human skin samples.

We have also produced a single-cell sequencing dataset, which has been made publicly available as a valuable resource for the community. While many studies have investigated diseased skin, and others have detailed the immune cell composition of healthy skin samples, there is a dearth of information at the single-cell level exploring the resident immune cell population of non-affected skin tissue from patients with skin cancer. We show that in this tissue, the sequential digestion method results in higher relative proportions of non-antigen-presenting mast cells, melanocytes, and CD8 T cells. Importantly, CD8 T cells resident in the skin play a critical role in autoimmune diseases [34], and they are thus of interest with regard to skin-related irAEs. The sequential digestion method also results in lower relative proportions of plasma cells, antigen-presenting mast cells, and KYNU^+^ CD4 T cells. Of note, the expression of KYNU (encoding kynureninase, a key enzyme in the kynurenine metabolic pathway involved in immune response) has been correlated with CD4 T cell state in the melanoma microenvironment [35]. The detection of CD4 T cells distinguished by KYNU expression suggests that gene expression changes resembling those seen in tumors may occur even in the photo-damaged adjacent skin of skin cancer patients, highlighting the importance of including such tissues as comparator samples.

Our study is necessarily limited by the institutional and informed consent restrictions on tissue collection, resulting in a lack of patient demographic and tissue resection site information for each sample. However, the division of each tissue sample prior to digestion with each of the tested methods should help to mitigate patient-to-patient bias, including bias stemming from patient demographics and sample resection site. Future studies should explicitly include and control for these factors when possible. 

Further work is needed to complement the steps we have taken toward elucidating the effect of tissue digestion methods on human skin samples. In addition to the examination of cell viability and yield presented here, an investigation of changes in cellular morphology will yield insights into potential changes in cellular function due to the choice of digestion method. Further high-throughput omics studies will also more completely characterize digestion method effects, including scRNA-seq studies examining differences in healthy, diseased, and non-affected human skin.

## 5. Conclusions

We set out to design an improved pipeline for single cell examination of human skin tissue. By testing variable digestion times and enzymes, we were able to analyze enriched immune cell populations within the skin with high viability and yield. We determined that the sequential enzyme digestion method yielded the best results on fresh tissue, which both maximizes viable cell yield and minimizes processing time. We demonstrate that a sequential digestion method is best to maximize the health and number of cells isolated from skin samples but also acknowledge the variation in isolated cell types between the sequential and simultaneous digestion methods. We showed significant differences in the relative proportions of various immune and non-immune cell types depending on the digestion method employed for tissue processing and have published these data as a resource for the community to aid in choosing the best skin digestion method for their study. 

## Data Availability

The data files are available through NCBI GEO at accession number GSE250390.

## Supplementary Materials

The following supporting information can be downloaded at https://www.mdpi.com/article/10.3390/cells13030266/s1, Table S1: AbSeq antibodies used to label surface proteins; Figure S1: Cell cluster annotation plots; Figure S2: Fresh vs. frozen sequencing quality control metrics; Table S2: Cell counts for digestion methods; Figure S3: Overnight digestion cell counts and viability compared to sequential and simultaneous digestions; Figure S4: Sequential vs. simultaneous digestion methods sequencing quality control metrics; Table S3: Differential proportion analysis: sequential vs. simultaneous digestions, CD45^+^ cell clusters; Table S4: Differential proportion analysis: sequential vs. simultaneous digestions, T cell subsets; Figure S5: scRNA-seq of CD45^−^ cells isolated from fresh tissue samples; Table S5: Differential proportion analysis: sequential vs. simultaneous digestions, CD45^−^ cell clusters.
