# Supplementary material for: Optimization of Tissue Digestion Methods for Characterization of Photoaged Skin by Single Cell RNA Sequencing Reveals Preferential Enrichment of T Cell Subsets
Source: Cells. 2024 Jan 31;13(3):266. doi: 10.3390/cells13030266 (PMC10854603; doi:10.3390/cells13030266)
Supplement: Supplementary file 1 [file cells-13-00266-s001.zip › cells-2823437-supplementary.pdf]

Supplemental Material

Table S1: BD AbSeq antibodies used to label surface proteins

Antibodies	Cat number	Seqeuence ID	Clone
CD117	940250	AHS0165	104D2
CD11b	940008	AHS0005	M1/70
CD11c	940024	AHS0056	B-LY6
CD138	940213	AHS0121	MI15
CD14	940257	AHS0173	M5E2
CD15	940274	AHS0196	W6D3
CD16	940006	AHS0053	3G8
CD161	940283	AHS0205	HP-3G10
CD19	940004	AHS0030	SJ25C1
CD193	940245	AHS0159	5E8
CD1a	940063	AHS0067	HI149
CD1c	940083	AHS0088	F10/21A3
CD1d	940296	AHS0219	CD1d42
CD206	940068	AHS0072	19.2
CD25	940463	AHS0166	M-A251
CD3	940000	AHS0033	SK7
CD4	940304	AHS0227	RPA-T4
CD45RA	940011	AHS0009	HI100
CD45RO	940022	AHS0036	UCHL1
CD56	940380	AHS0257	B159
CD62L	940387	AHS0265	SK11
CD68	460076	AHS0293	Y1/82A
CD69	940019	AHS0010	FN50
CD8	940305	AHS0228	SK1
CD80	940036	AHS0046	L307.4
CD86	940025	AHS0057	2331 (FUN-1)
FCER1A	940220	AHS0129	AER-37
HLA-DR	940010	AHS0035	G46-6
LAG3	940080	AHS0018	T47-530
PD1 (CD279)	940467	AHS0190	MIH4
TCRgd	940057	AHS0015	B1
TIM-3	940066	AHS0016	7D3
Vα7.2	460065	AHS028Q	OF-5A12

Figure S1: Cell cluster annotation plots

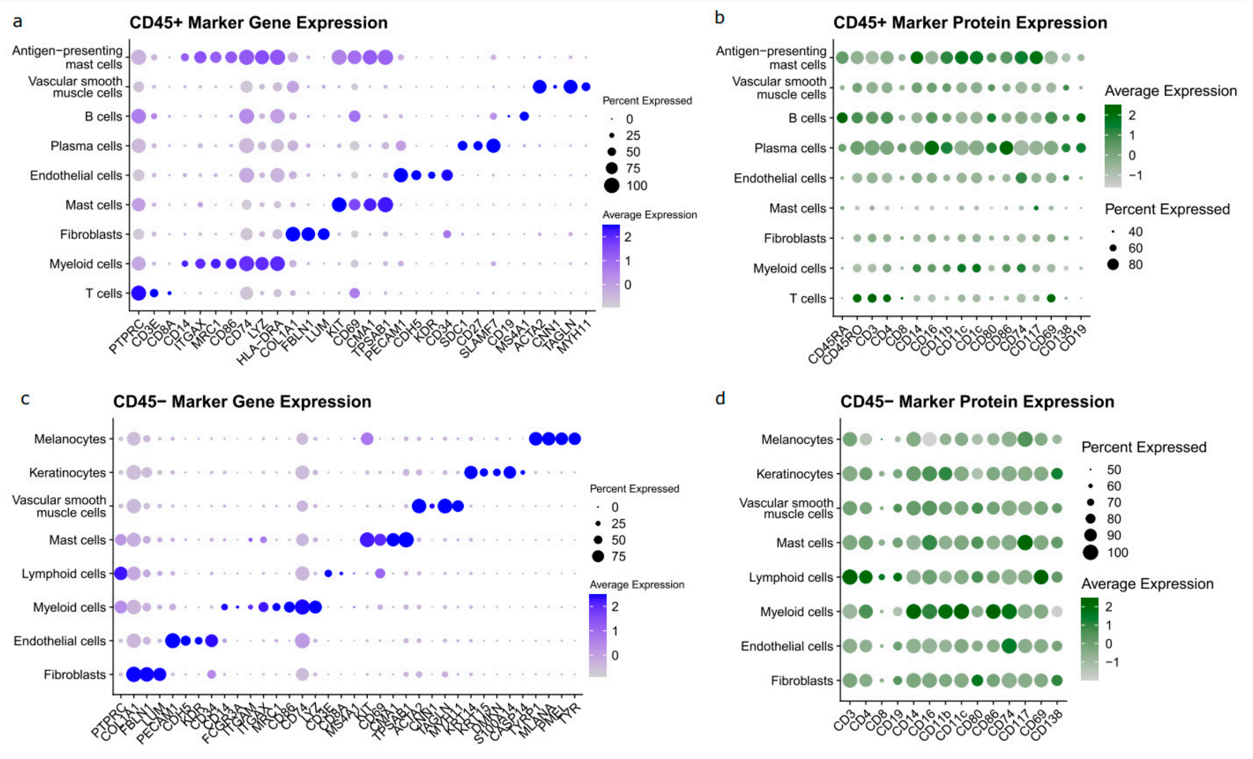

Figure S1: Cell cluster annotation plots. (a) Dot plot shows expression of genes used to annotate CD45^{+} cell clusters. Dots are colored by average relative gene expression of cells in each cluster, and sized by the percent of cells expressing each gene. (b) Dot plot shows expression of proteins used to annotate CD45^{+} cell clusters. Dots are colored by average relative protein expression of cells in each cluster, and sized by the percent of cells expressing each protein. (c) Same as (a), but for CD45^{-} cell clusters. (d) Same as (b), but for CD45^{-} cell clusters.

Figure S2: Fresh vs. frozen sequencing quality control metrics

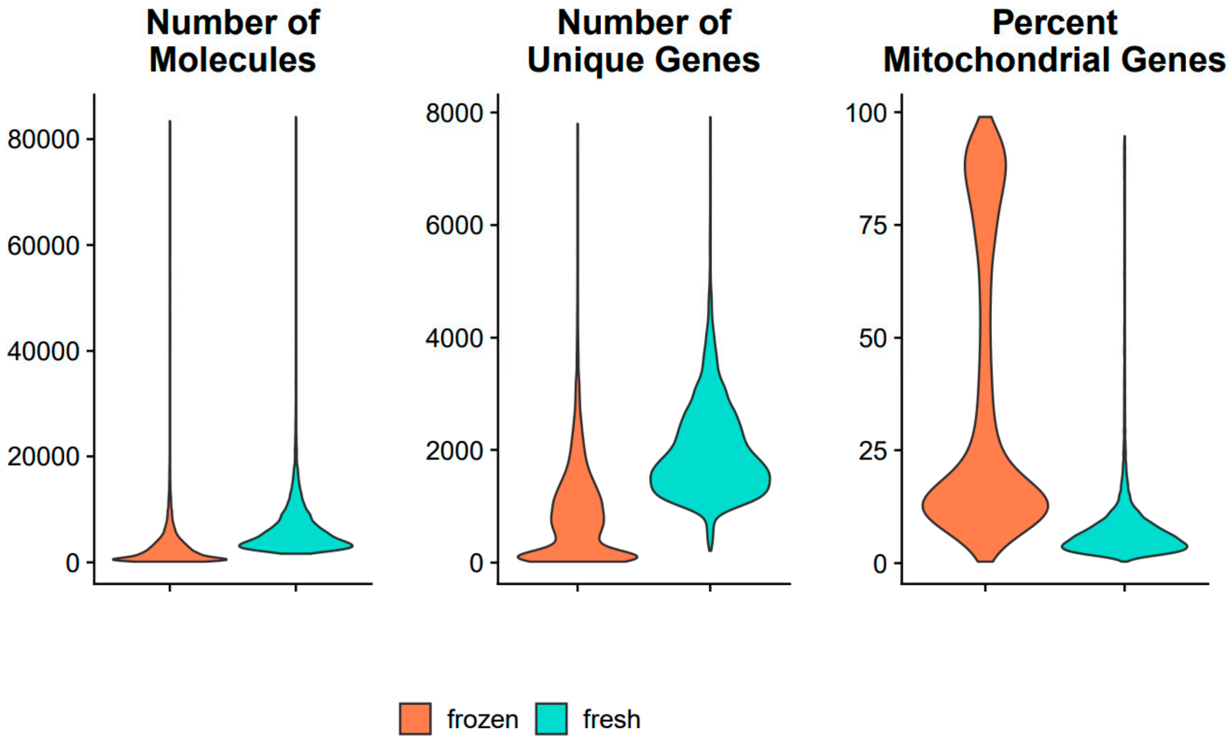

Figure S2: Fresh vs. frozen sequencing quality control metrics. Violin plots show the distribution among all CD45^{+} cells processed using the simultaneous digestion method of the number of molecules detected (left panel), number of unique genes detected (middle panel), and the percent of mitochondrial genes detected (right panel). Frozen (coral) and fresh (turquoise) tissue states are compared.

Figure S3: Overnight digestion cell counts and viability compared to sequential and simultaneous digestions

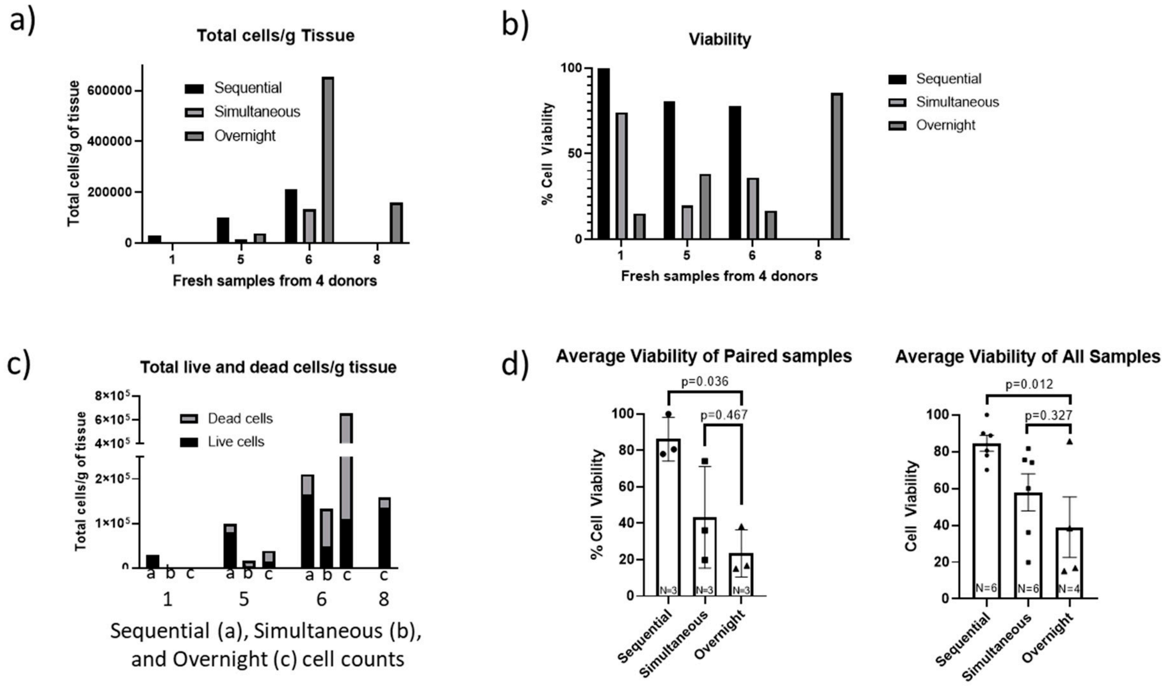

Figure S3: Overnight digestion method cell counts and viability. (a) Total cells per gram of tissue for three patients whose tissue was sizable enough to cut into 3 roughly 4mm sections and digest using all three (sequential, simultaneous, and overnight) digestion methods (patients #1, 5, and 6). Also shown is an additional patient (#8) whose tissue was digested exclusively with the overnight method. (b) Cell viability for the same samples from (a) are shown. (c) Overnight digestion method of live and dead cell counts for each total cell count shown in part (a). (d) Average cell viability of both the paired samples (N=3, patients #1, 5, and 6) with paired t-tests demonstrating significant difference between sequential and overnight digestion methods (p=0.036) but not between simultaneous and overnight digestion methods (p=0.467) (left). The average viability for all samples is also shown (right) and there is likewise a statistically significant difference between the sequential and overnight digestion methods (unpaired t-test, p=0.012) but not between the simultaneous and overnight methods (unpaired t-test, p=0.327). Error bars indicate SEM.

Table S2: Cell counts for digestion methods

Sample	Weight (g)	Digestion Approach	Viability	Total Cells	Total cells/g of Tissue
**1a**	0.2516	sequential	100	28,012	111,335
**1b**	0.2613	simultaneous	74.14	1,652	6,322
**1c**	0.2327	overnight	15.15	1,440	6,188
**2a**	0.2261	sequential	89.95	323,506	1,430,809
**2b**	0.2791	simultaneous	81.7	264,586	947,997
**3a**	0.0688	sequential	88.82	60,876	884,826
**3b**	0.0441	simultaneous	75.55	168,130	3,812,472
**4a**	0.1406	sequential	70.15	161,308	1,147,283
**4b**	0.1305	simultaneous	60.07	104,584	801,410
**5a**	0.2904	sequential	80.64	122,964	423,430
**5b**	0.2016	simultaneous	19.85	77,880	386,310
**5c**	0.272	overnight	38.35	100,226	368,478
**6a**	0.2464	sequential	78	51,853	210,441
**6b**	0.2427	simultaneous	36.1	32,402	133,505
**6c**	0.2146	overnight	16.81	140,178	653,208
**7a**	0.0281	sequential	60.83	18,439	656,199
**7b**	0.0291	simultaneous	14.34	10,637	365,522
**8**	0.4948	overnight	85.61	184,450	372,777

Figure S4: Sequential vs. simultaneous digestion methods sequencing quality control metrics

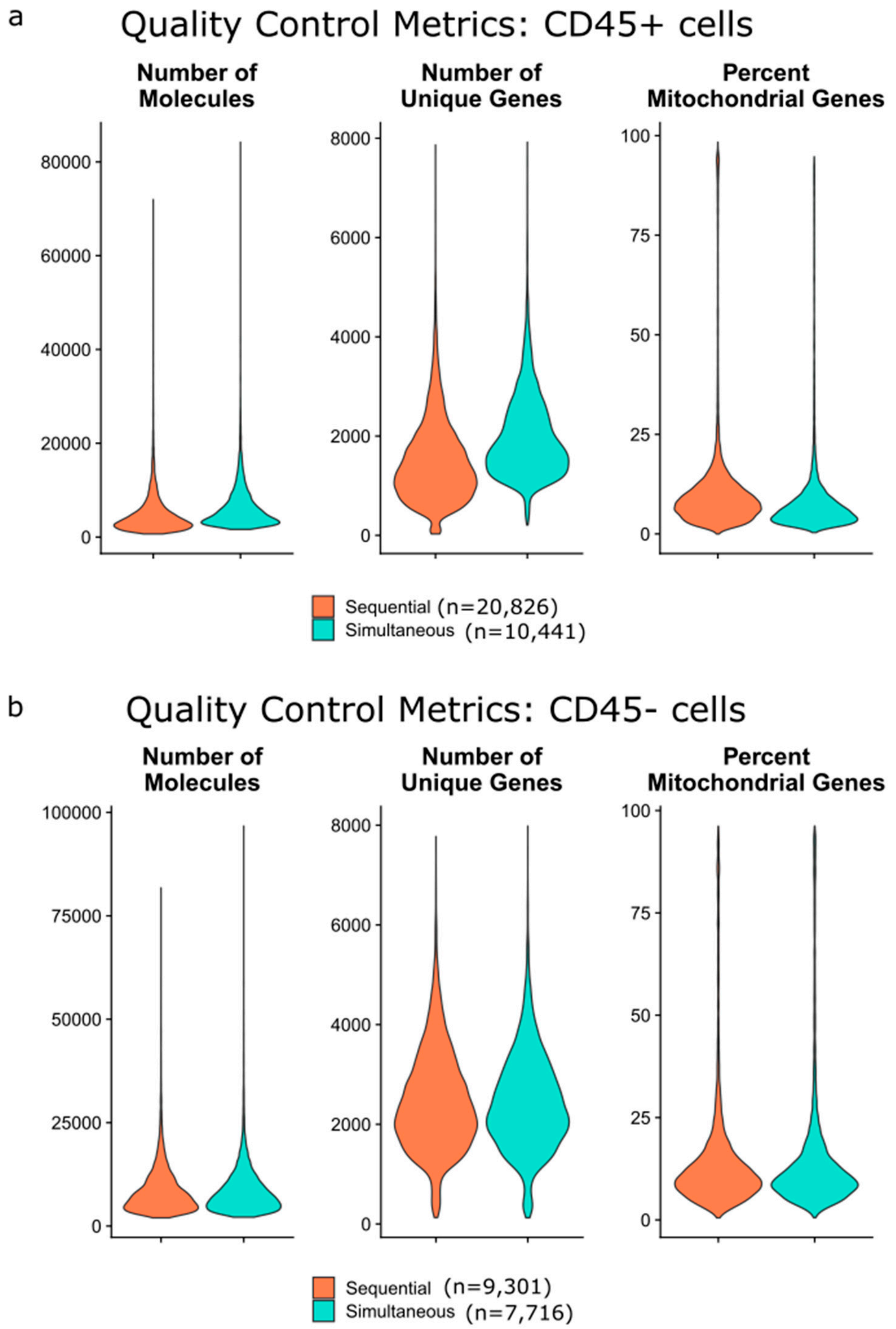

Figure S4: Sequencing quality control metrics for sequential and simultaneous digestion. (a) Violin plots show the distribution among all CD45^{+} cells processed from fresh tissue of the number of molecules detected (left panel), number of unique genes detected (middle panel), and the percent of mitochondrial genes detected (right panel). Sequential (coral) and simultaneous (turquoise) digestion methods are compared. Number of cells per digestion method is reported in parentheses in the legend. (b) As (a), but for CD45^{-} cells.

Figure S5: scRNA-seq of CD45$^-$ cells isolated from fresh tissue samples

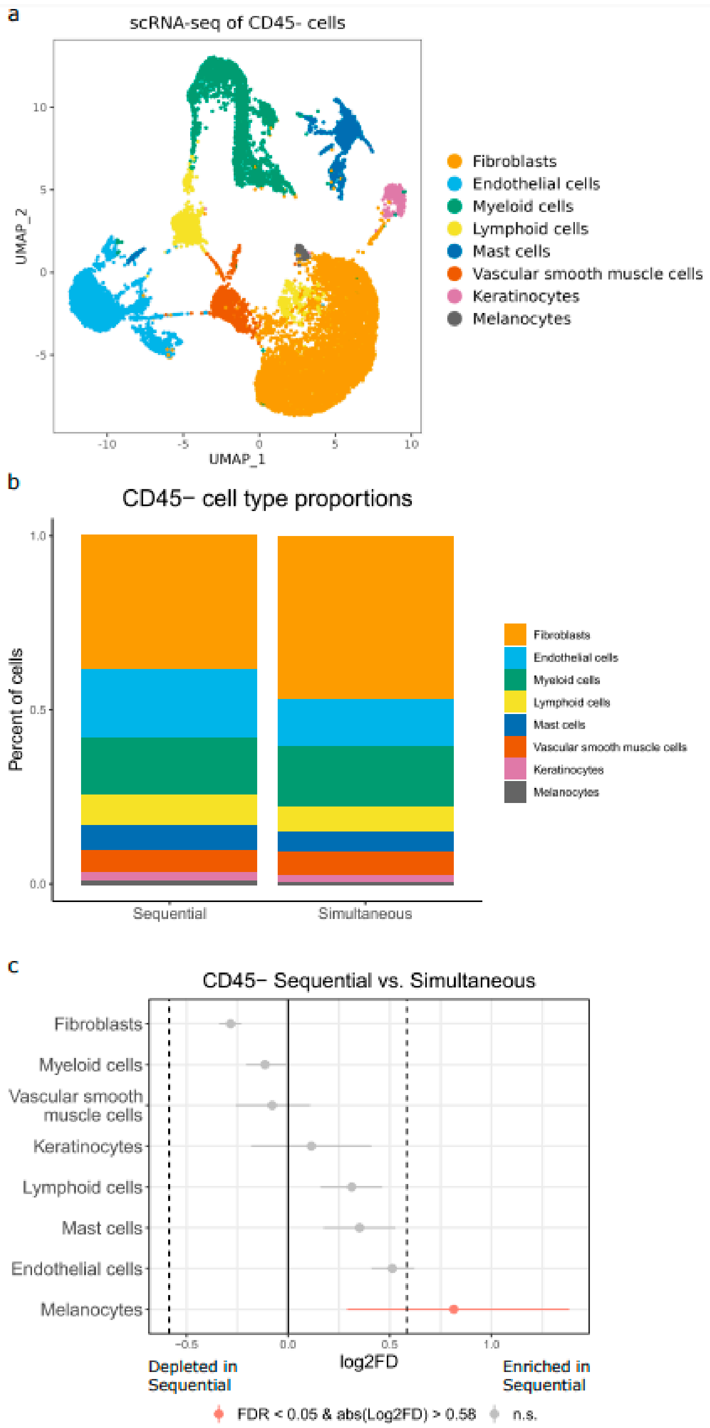

Figure S5: scRNA-seq of CD45$^-$ cells isolated from fresh tissue samples. (a) UMAP plot of 15,300 annotated CD45$^-$ cells colored by cell type. (b) Stacked bar plot showing the relative proportions of each CD45$^-$ cell type, split by digestion method. (c) Relative differences in cell proportions for each CD45$^-$ cluster, comparing sequential and simultaneous digestion methods. Red clusters are statistically significant (FDR < 0.05 and absolute $\log_2$ fold change > 0.58). Larger $\log_2$ fold changes indicate a higher proportion of cells in the sequential method

Table S3: Differential proportion analysis: sequential vs. simultaneous digestions, CD45^{+} cell clusters

Clusters	Sequential	Simultaneous	obs_log2FD	pval	FDR	boot_mean_log2FD	boot_CI_2.5	boot_CI_97.5
Antigen-presenting mast cells	0.001726765	0.003816411	-1.144144748	0.000999001	0.001123876	-1.14470096	-1.831676983	-0.452914018
B cells	0.0107161	0.013257005	-0.306975007	0.028971029	0.028971029	-0.308087727	-0.614446266	0.004547486
Endothelial cells	0.012442864	0.019684644	-0.661751981	0.000999001	0.001123876	-0.666320898	-0.915749014	-0.411450993
Fibroblasts	0.050228542	0.07893944	-0.652238867	0.000999001	0.001123876	-0.65208555	-0.778059955	-0.525078627
Mast cells	0.054850178	0.035251582	0.637808301	0.000999001	0.001123876	0.6373523	0.466202797	0.814537156
Myeloid cells	0.170543423	0.222556995	-0.384035727	0.000999001	0.001123876	-0.382544757	-0.451193514	-0.315727169
Plasma cells	0.008329101	0.021492417	-1.367595058	0.000999001	0.001123876	-1.369348605	-1.659274075	-1.068144843
T cells	0.685068563	0.589334137	0.217162547	0.000999001	0.001123876	0.216812804	0.190006099	0.245150496
Vascular smooth muscle cells	0.006094464	0.01566737	-1.362191699	0.000999001	0.001123876	-1.354060985	-1.706166716	-1.00504211

Table S4: Differential proportion analysis: sequential vs. simultaneous digestions, T cell subsets

clusters	Sequential	Simultaneous	obs_log2FD	pval	FDR	boot_mean_log2FD	boot_CI_2.5	boot_CI_97.5
ANXA1+ CD4 T cells	0.61657647	0.548909339	0.167711949	0.000999001	0.000999001	0.168577261	0.130907016	0.205290628
CD8 T cells	0.125732078	0.077027948	0.706898869	0.000999001	0.000999001	0.708577948	0.56174146	0.850785002
KYNU+ CD4 T cells	0.123730447	0.201772324	-0.705527749	0.000999001	0.000999001	-0.705648696	-0.800370569	-0.607924741
Regulatory T cells	0.133961005	0.172290389	-0.363029114	0.000999001	0.000999001	-0.365888966	-0.473066474	-0.263041256

Table S5: Differential proportion analysis: sequential vs. simultaneous digestions, CD45^{-} cell clusters

clusters	Sequential	Simultaneous	obs_log2FD	pval	FDR	boot_mean_log2FD	boot_CI_2.5	boot_CI_97.5
Endothelial cells	0.197871832	0.138696655	0.512633255	0.000999001	0.001998002	0.515356024	0.408680935	0.619959344
Fibroblasts	0.382592061	0.465397924	-0.282657837	0.000999001	0.001998002	-0.282622389	-0.338819316	-0.230615015
Keratinocytes	0.024509804	0.022635525	0.114770187	0.244755245	0.244755245	0.110646293	-0.181273453	0.409244429
Lymphoid cells	0.091343855	0.073529412	0.312986228	0.000999001	0.001998002	0.313018682	0.158668104	0.462242048
Mast cells	0.073409852	0.057525952	0.351760746	0.000999001	0.001998002	0.346258467	0.17412362	0.526786847
Melanocytes	0.010401722	0.005911188	0.815302328	0.001998002	0.003196803	0.827426642	0.290113606	1.382496249
Myeloid cells	0.160329986	0.173442907	-0.113416569	0.015984016	0.021312021	-0.112952096	-0.20781037	-0.008462396
Vascular smooth muscle cells	0.05954089	0.062860438	-0.078271556	0.203796204	0.232909947	-0.077944006	-0.258042715	0.109007684
